# Exploring the potential mechanism of Simiao Yongan decoction in the treatment of diabetic peripheral vascular disease based on network pharmacology and molecular docking technology

**DOI:** 10.1097/MD.0000000000036762

**Published:** 2023-12-29

**Authors:** Fang Cao, Yongkang Zhang, Yuan Zong, Xia Feng, Junlin Deng, Yuzhen Wang, Yemin Cao

**Affiliations:** a Shanghai University of Traditional Chinese Medicine, Shanghai, China; b Diagnosis and Treatment Center of Vascular Disease, Shanghai TCM-Integrated Hospital, Shanghai University of Traditional Chinese Medicine, Shanghai, China.

**Keywords:** diabetic peripheral vascular disease, molecular docking, network pharmacology, Simiao Yongan decoction

## Abstract

The study aims to investigate the potential action targets and molecular mechanisms of Simiao Yongan decoction (SMYAD) in treating diabetic peripheral vascular disease (DPVD) by utilizing network pharmacology analysis and molecular docking technology. The components and targets of SMYAD were screened using the TCMSP database, while DPVD-related genes were obtained from the GeneCards, OMIM, and Disgenet databases. After intersecting the gene sets, a Protein-Protein Interaction (PPI) network was established, and Gene Ontology (GO) and Kyoto Encyclopedia of Genes and Genomes (KEGG) enrichment analyses were carried out. The practical chemical components and core targets identified were molecularly docked using AutoDock software. A total of 126 active compounds were screened from which 25 main components included quercetin, rutoside, hesperidin, naringin, and β-sitosterol were determined to be the active components most associated with the core targets. A total of 224 common target genes were obtained. Among them, JUN, AKT1, MAPK3, TP53, STAT3, RELA, MAPK1, FOS, and others are the expected core targets of traditional Chinese medicine. The top-ranked GO enrichment analysis results included 727 biological processes (BP), 153 molecular functions (MF), and 102 cellular components (CC). KEGG pathway enrichment analysis involved mainly 178 signaling pathways, such as cancer signaling pathway, AGE-RAGE signaling pathway, interleukin-17 signaling pathway, tumor necrosis factor signaling pathway, endocrine resistance signaling pathway, cell aging signaling pathway, and so on. The molecular docking results demonstrate that the principal chemical components of SMYAD exhibit considerable potential for binding to the core targets. SMYAD has the potential to treat DPVD through various components, targets, and pathways. Its mechanism of action requires further experimental investigation.

## 1. Introduction

Diabetic Peripheral Vascular Disease (DPVD) is a common complication of diabetes, characterized as a chronic ischemic disease caused by atherosclerosis in the peripheral arteries over the course of long-term pathological changes.^[1]^ Diabetes is a global public health challenge, with an estimated 51% increase in the number of diabetic patients worldwide by 2045, reaching 700 million. Among these, approximately 9.5% of diabetic patients over the age of 40 suffer from peripheral vascular disease, and the incidence of peripheral vascular disease in patients with diabetes for 5 years or more is as high as 90%.^[2,3]^

The typical clinical manifestations of DPVD include dry skin, abnormal limb sensation, and decreased skin temperature. In severe cases, it is accompanied by intermittent claudication or rest pain, eventually leading to limb gangrene, local tissue necrosis infection, and even amputation.^[4]^

DPVD leading to limb gangrene infection has become one of the significant causes of disability in diabetic patients. Statistics show that the amputation rate in patients with diabetes complicated by peripheral vascular disease reaches 26%, which is 5-10 times that of the normal population.^[5,6]^ Modern medical treatment for early DPVD primarily involves controlling the primary disease, such as controlling blood sugar, antithrombotic therapy, lipid-lowering, and antihypertensive therapy. This is supplemented with spinal cord stimulation, pneumatic compression, prostaglandin drugs, and hyperbaric oxygen, but their effectiveness is not yet clear.^[7]^ Patients with moderate to high-risk limb ischemic gangrene should improve blood circulation and control infection early. Treatment plans include vascular reconstruction, oral or intravenous antibiotics, and instrument debridement. However, antibiotics and anticoagulants can lead to drug resistance and coagulation disorders, making the search for more effective, safer, and less side-effect-prone alternative therapies particularly important.

Simiao Yongan decoction (SMYAD) is a traditional Chinese medicine formula that was initially documented in “Shen Yi Mi Zhuan” by Hua Tuo. It contains Angelica, Honeysuckle, Scrophularia, and Licorice. The formula’s benefits include clearing heat, detoxifying, promoting blood circulation, and relieving pain, making it commonly used for heat-toxin accumulation syndrome. It can effectively alleviate vascular inflammation, achieving anti-inflammatory, analgesic, lipid-lowering, and antithrombotic effects,^[8–11]^ with definite therapeutic efficacy. However, a very small number of patients in the SMYAD group experienced adverse effects such as nausea, vomiting and dizziness in the clinical trial, but the incidence was much lower than in the Western medicine group.^[12,13]^

However, SMYAD belongs to the traditional Chinese medicine compound formula, in which the drug composition is complex, and the mechanism of action for the treatment of DPVD is not completely unclear. Accordingly, this examination employs network pharmacology and molecular docking technology to foretell and preliminarily substantiate SMYAD’s mechanism of action in DPVD treatment, thus providing a clear direction for future basic research.

## 2. Data and methods

### 2.1. Databases and software

The databases and software used in this study include The TCMSP (https://tcmsp-e.com/tcmsp.php),^[14]^ PubChem(https://pubchem.ncbi.nlm.nih.gov/),^[15]^ UniProt (https://www.uniprot.org/),^[16]^String(https://string-db.org/),^[17]^GeneCards (https://www.genecards.org/),^[18]^ Online Mendelian Inheritance in Man (OMIM) (https://omim.org/),Disgenet (https://www.disgenet.org/),PDB (https://www.rcsb.org/), The Protein–ligand interaction profiler database(PLIP) (https://plip.biotec.tu-dresden.de/plip-web/plip/index),^[19]^David databases (https://david.ncifcrf.gov/),^[20]^ *Cytoscape 3.8.0,*^[21]^ *OpenBabel-2.4.1,*^[22]^ *Autoduck vina 1.5.6*,^[23]^ *Pymol 2.5.4,*^[24]^ *Pycharm 2022.2.2*, and Weishengxin (http://www.bioinformatics.com.cn).

### 2.2. Collection and treatment of Simiao Yongan decoction targets

The keywords “Honeysuckle,” “Scrophularia,” “Angelica,” and “Licorice” were used to search in the Traditional Chinese Medicine Systems Pharmacology analysis platform (https://tcmsp-e.com/tcmsp.php TCMSP).^[14]^ The effective chemical components of the 4 herbs were searched and screened according to the standards of Oral Bioavailability (OB) > 30% and Drug-Likeness (DL) > 0.18. After removing duplicates, the effective chemical components of SMYAD and their corresponding action targets were predicted. Finally, the “Homo sapiens” species was selected on the Uniprot database platform (https://www.uniprot.org/),^[16]^ and the screened protein targets were standardized.

### 2.3. Identification of target genes related to DPVD

The keyword “Diabetic Peripheral Vascular Disease” was used to search for related disease targets of DPVD in the GeneCards databases, Online Mendelian Inheritance in Man (OMIM) and Disgenet databases.^[18]^ The data from the 3 databases were merged and duplicates were removed to obtain a collection of DPVD disease targets.

### 2.4. Goals related to treatment of DPVD with SMYAD

The disease targets obtained from the above text and the targets of the effective chemical components of the drug were intersected to obtain the common targets of SMYAD and DPVD.

### 2.5. Construction of the protein-protein interaction network

The common targets of the drug and disease were imported into the STRING database (https://cn.string-db.org/).^[17]^ The function was set to “Multiple proteins,” the species was set to “Homo sapiens,” the “highest confidence” was set to ≥ 0.9, and “hide disconnected nodes in the network” was selected to hide free nodes. This resulted in a Protein–protein interaction (PPI). The predicted protein interaction relationships were saved in TSV format and imported into *Cytoscape 3.8.0* software^[22]^ for network topology analysis.

### 2.6. Drug-active component-target gene-disease network

The core disease targets of TCM, active ingredients of TCM, and TCM were organized into network files and attribute files, respectively, and uploaded to

*Cytoscape 3.8.0* software to construct a network diagram of the core action targets of SMYD-Active Ingredients-DPVD. Network parameter analysis was performed using the Network Analyzer tool in *Cytoscape 3.8.0* software to derive the main active ingredients and key targets to further explore the molecule-target relationship of DPVD treatment with SMYAD.

### 2.7. GO and KEGG enrichment analysis

The common targets of the drug and disease were uploaded to the David database^[21]^ for gene ontology (GO) and Kyoto Encyclopedia of Genes and Genomes (KEGG) pathway enrichment analysis. The analysis results were screened according to the standards of *P* ≤ .05 and FDR ≤ 0.05. The top 20 results with higher enrichment levels were uploaded to the Weishengxin online drawing platform (http://www.bioinformatics.com.cn). The results were displayed in bar and strip charts.

### 2.8. Molecular docking

The main effective active components screened out from the “Traditional Chinese Medicine-Effective Active Components-Key Disease” were selected as ligand small molecules, and the common core targets of the drug and disease were used as protein molecules. The SDF files were downloaded from the PubChem database^[15]^ (http://pubchem.ncbi.nlm.nih.gov/) and imported into *OpenBabel-2.4.1*^[22]^ to be converted into mol2 format for export. The target protein 3D structure was searched and downloaded from the PDB database(https://www.rcsb.org/). Nonpolar hydrogen was added using *AutoDock 1.5.6* software.^[23]^ Finally, the *AutoDock Vina* program was used to calculate the binding energy between the target protein and the main blood-activating components, and the receptor was visualized using *Pymol* software^[24]^ and the Protein-Ligand Interaction Profiler database.^[20]^

## 3. Results

### 3.1. Research roadmap

**Figure 1.**
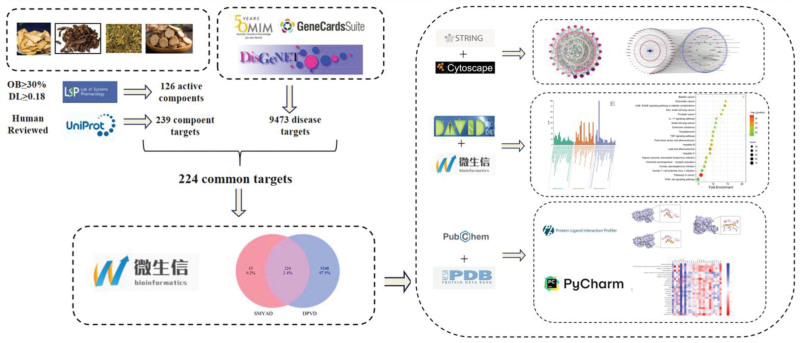
Research roadmap.

The literature elucidated the effectiveness of SMYAD against DPVD. TCMSP determined the active ingredients of SMYAD and predicted their corresponding targets, while Uniprot standardized the annotation of those targets. The Genecard, OMIM, and Disgenet databases determined the collection of DPVD disease targets. The intersection targets of SMYAD and DPVD databases were obtained using a Venn diagram. The potential core targets were confirmed by constructing PPIs through the STRING database utilizing the above-stated targets. To determine key compounds that exert therapeutic effects, a database comprising SMYAD key compounds and DPVD disease targets was created. Network topology analysis was performed using *Cytoscape 3.8.0*. The targets of SMYAD for DPVD underwent GO enrichment and KEGG enrichment analysis after being imported into the DAVID database. The compound SDF structures were acquired from the PubChem database, while the protein SDF structures were obtained from the RCSB database in PDB files. Molecular docking was carried out utilizing *Autodock* software, and the validation outcomes were displayed using *PyMOL* software and the PLIP database. The procedure is illustrated in Figure 1.

### 3.2. The collection and screening of Simiao Yongan decoction active ingredients

A total of 239 protein targets were gathered after screening 126 effective chemical components of SMYAD from the TCMSP database, which adhered to the criteria of Oral Bioavailability (OB) > 30% and Drug-Likeness (DL) > 0.18. Uniprot database (https://www.uniprot.org/) was utilized to standardize the protein targets obtained from Honeysuckle, Licorice, Angelica, and Scrophularia, acquiring 449 targets, 1769 targets, 69 targets, and 68 targets, respectively. Finally, duplicates were removed to obtain the 239 protein targets.

The TCMSP database was searched for compounds with an OB > 30% and DL > 0.18, leading to the identification of 126 active chemical components in SMYAD. Target prediction identified 449 targets for honeysuckle, 1769 for licorice, 69 for angelica, and 68 for figwort root, and 239 protein targets were finally obtained after deleting duplicate values. Following the removal of duplicates, the final dataset consisted of 239 protein targets that were normalized using the Uniprot database (https://www.uniprot.org/).

### 3.3. Screening of target proteins of the active ingredients of DPVD

**Figure 2.**
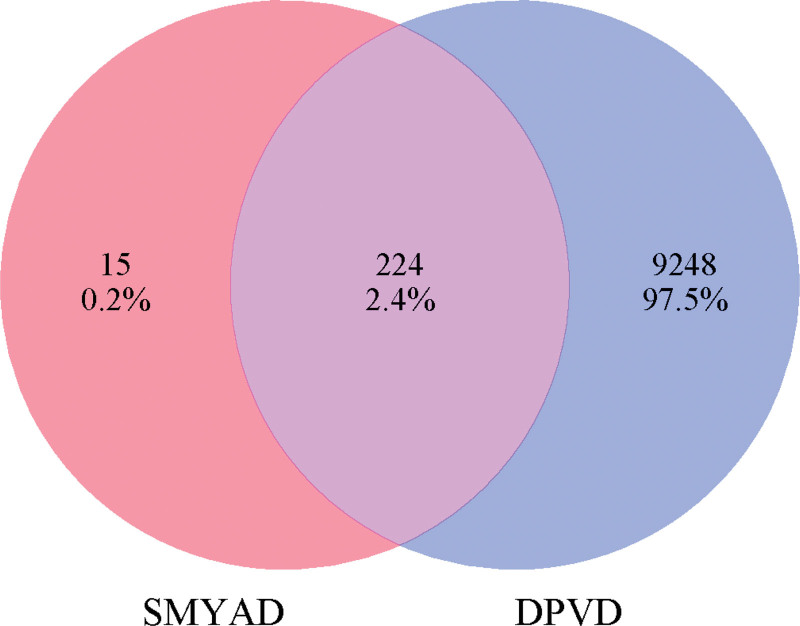
Venn diagram for “SYMTD component-target” and “DPVD disease-target” intersection. DPVD = diabetic peripheral vascular disease, SMTAD = Simiao Yongan decoction.

From the GeneCards, OMIM, and Disgenet databases, we identified 8424, 1394, and 1 targets for DPVD disease respectively. After merging genes from these databases and removing duplicates, a total of 9473 DPVD disease targets were identified. As a result, we identified 224 potential protein targets for DPVD treatment using SMYAD. The Weishengxin online drawing tool(http://www.bioinformatics.com.cn) was utilized to generate a Venn diagram depicting the overlap between drug and disease target genes, demonstrated in Figure 2

### 3.4. Construction of the protein-protein interaction network

**Figure 3.**
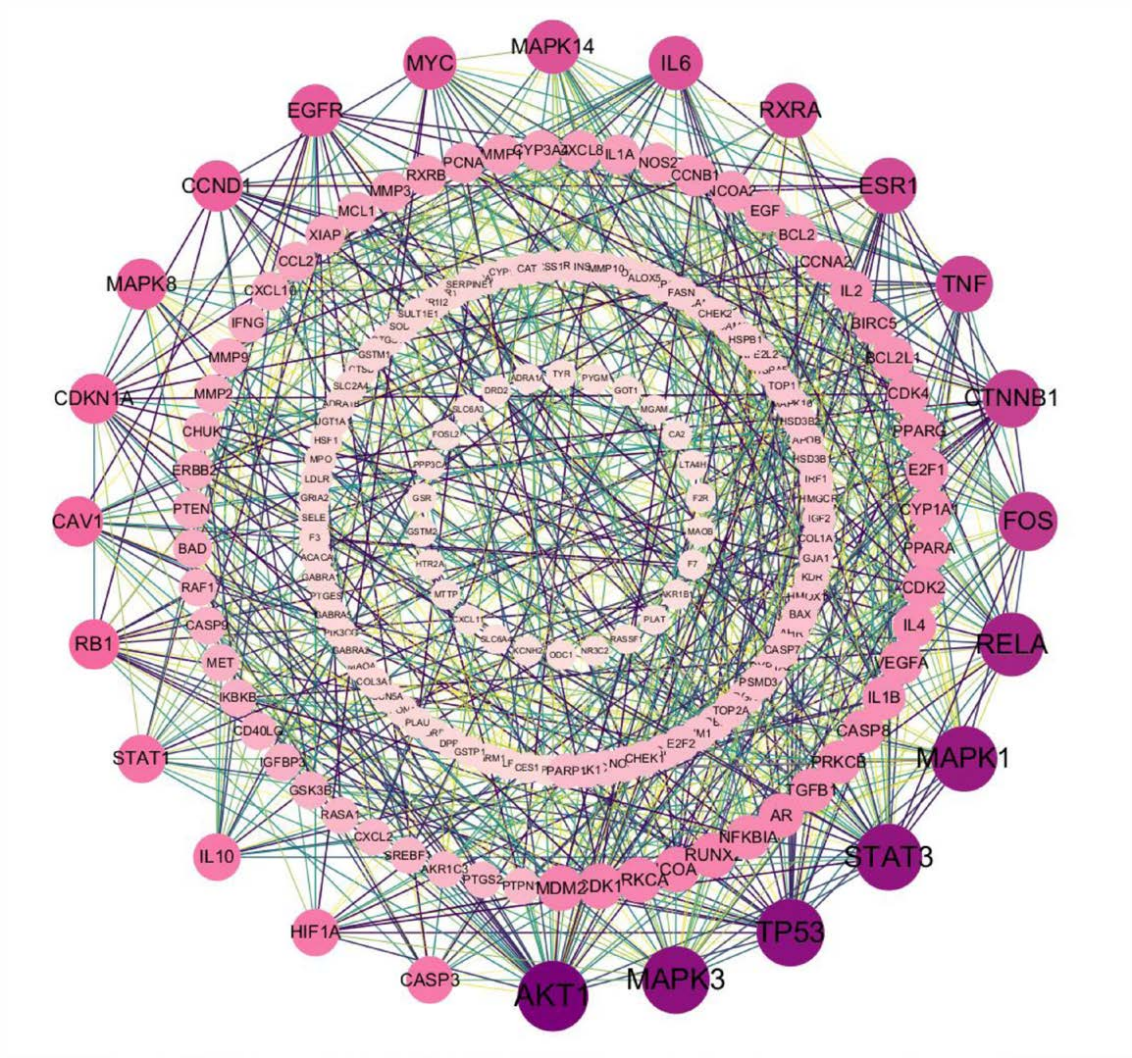
The PPI network of 224 target genes. The degree indicates the number of interactions with the target. Targets with larger and darker nodes play a more significant role. The outermost layer is vital in the PPI network. The combined score indicates the strength of the interaction between 2 nodes. The darker and thicker the line, the stronger the interaction between the 2 targets.

The core targets of SMYAD for treating DPVD were uploaded to the String database, with removal of free nodes and confidence level set to 0.09. The protein interaction information was screened and then imported into *Cytoscape 3.8.0* software to create the PPI diagram illustrated in Figure 3. The diagram includes 189 nodes and 867 lines, with the size of each node indicating the degree of objective relationship. The average degree value of the nodes is 9.175. Core nodes were screened from values exceeding twice the average degree. The distribution is depicted based on the degree value’s size, as presented in Figure 3. Core targets with a Degree value greater than 18, such as AKT1, MAPK3, TP53, STAT3, MAPK1, RELA, FOS, CTNNB1, TNF, ESR1, RXRA, MAPK14, IL-6, MYC, EGFR, CCND1, MAPK8, RB1, CAV1, CDKN1A, STAT1, CASP3, HIF1A, IL-10, and others, may have an important role in the PPI network based on Table 1.

**Table 1:** The core target proteins of SMYAD in the treatment of DPVD.

Number	Target name	Degree
1	AKT1	43
2	MAPK3	40
3	TP53	40
4	STAT3	39
5	MAPK1	38
6	RELA	36
7	FOS	31
8	CTNNB1	30
9	TNF	29
10	ESR1	28
11	RXRA	27
12	MAPK14	26
13	IL-6	26
14	MYC	25
15	EGFR	24
16	CCND1	23
17	MAPK8	23
18	RB1	22
19	CAV1	22
20	CDKN1A	22
21	STAT1	20
22	CASP3	19
23	HIF1A	19
24	IL-10	19

DPVD = diabetic peripheral vascular disease, SMYAD = Simiao Yongan Decoction.

### 3.5. Create the “active component-DPVD disease target” network diagram

**Figure 4.**
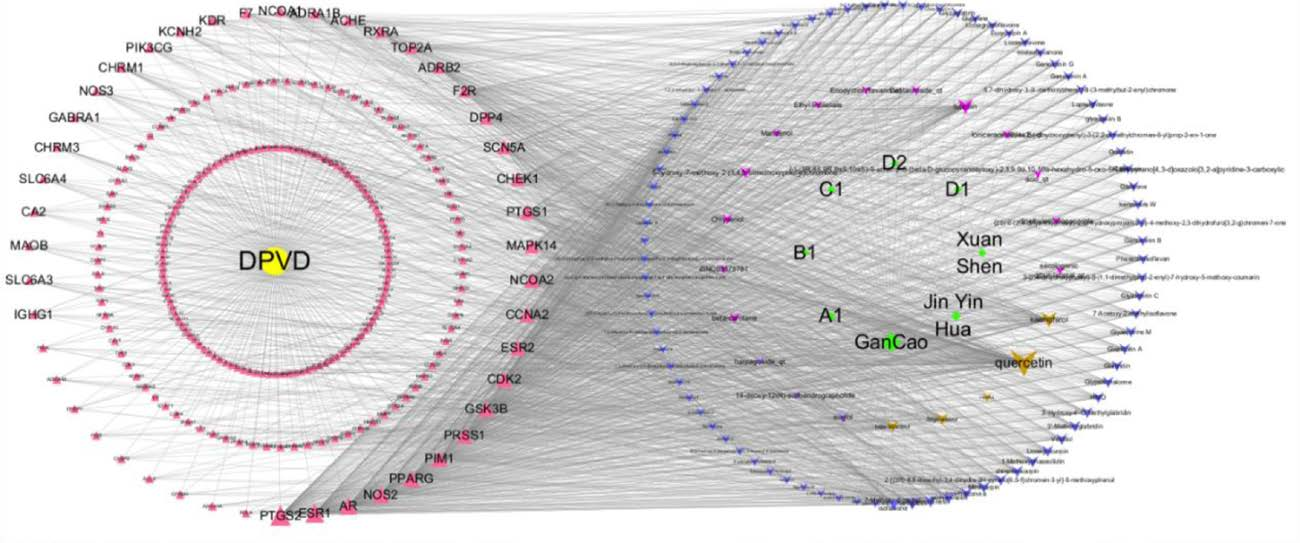
Medicine-active ingredients-target-DPVD target network. Δrepresents the intersection targets of the drug and disease (DPVD target),∇ represents the effective components of the drug (Active components), • represents the DPVD disease, ⋄represents the SMYAD formula. Among them, A1 represents Dang Gui, Xuan Shen, and Jin Yin Hua; B1 represents Dang Gui and Jin Yin Hua; C1 represents Xuan Shen and Gan Cao; D1 and D2 represent Jin Yin Hua and Gan Cao. DPVD = diabetic peripheral vascular disease, SMYAD = Simiao Yongan decoction.

SMYAD, including its effective chemical components and disease core targets, was used to construct a network diagram on *Cytoscape 3.8.0* software. Figure 4 displays that there is a total of 334 nodes in the diagram, containing 224 core targets, 4 traditional Chinese medicines, and 106 effective active components. Upon conducting network topology analysis on the data, the larger the node, the higher the degree value, and the more significant its key role in the network. The average degree value of the nodes is 11.16. We screened for core nodes using values twice the average degree as our criteria.^[17]^ Our findings indicate that 25 compounds, including quercetin, carnosol, osajin, 7-methoxy-2-methyl isoflavone, naringenin, and β-sitosterol, are the main chemical components of SMYAD, as depicted in Table 2.

**Table 2:** The main chemical components of SMYAD for the treatment of DPVD.

Number	Medicine	Molecule name	Degree
1	D1	quercetin	141
2	D2	kaempferol	57
3	Jin Yin Hua	luteolin	53
4	GanCao	7-Methoxy-2-methyl isoflavone	39
5	GanCao	naringenin	36
6	GanCao	Formononetin	33
7	GanCao	Isorhamnetin	33
8	A1	beta-sitosterol	33
9	GanCao	Licochalcone A	30
10	GanCao	Medicarpin	30
11	B1	Stigmasterol	30
12	GanCao	2-[(3R)-8,8-dimethyl-3,4-dihydro-2H-pyrano[6,5-f]chromen-3-yl]-5-methoxyphenol	29
13	GanCao	shinpterocarpin	28
14	GanCao	Licoagrocarpin	27
15	GanCao	Vestitol	27
16	GanCao	1-Methoxyphaseollidin	27
17	GanCao	3′-Methoxyglabridin	26
18	GanCao	3′-Hydroxy-4′-O-Methylglabridin	25
19	GanCao	HMO	25
20	GanCao	Glabridin	24
21	GanCao	Glypallichalcone	24
22	GanCao	Glyasperins M	23
23	GanCao	7-Acetoxy-2-methylisoflavone	23
24	GanCao	Glepidotin A	23
25	Jin Yin Hua	5-hydroxy-7-methoxy-2-(3,4,5-trimethoxyphenyl)chromone	23

A1 = Dang Gui, Xuan Shen, and Jin Yin Hua, B1 = Dang Gui and Jin Yin Hua, D1 = Jin Yin Hua and Gan Cao, D2 = Jin Yin Hua and Gan Cao, DPVD = diabetic peripheral vascular disease, SMYAD = Simiao Yongan decoction.

### 3.6. GO enrichment analysis

**Figure 5.**
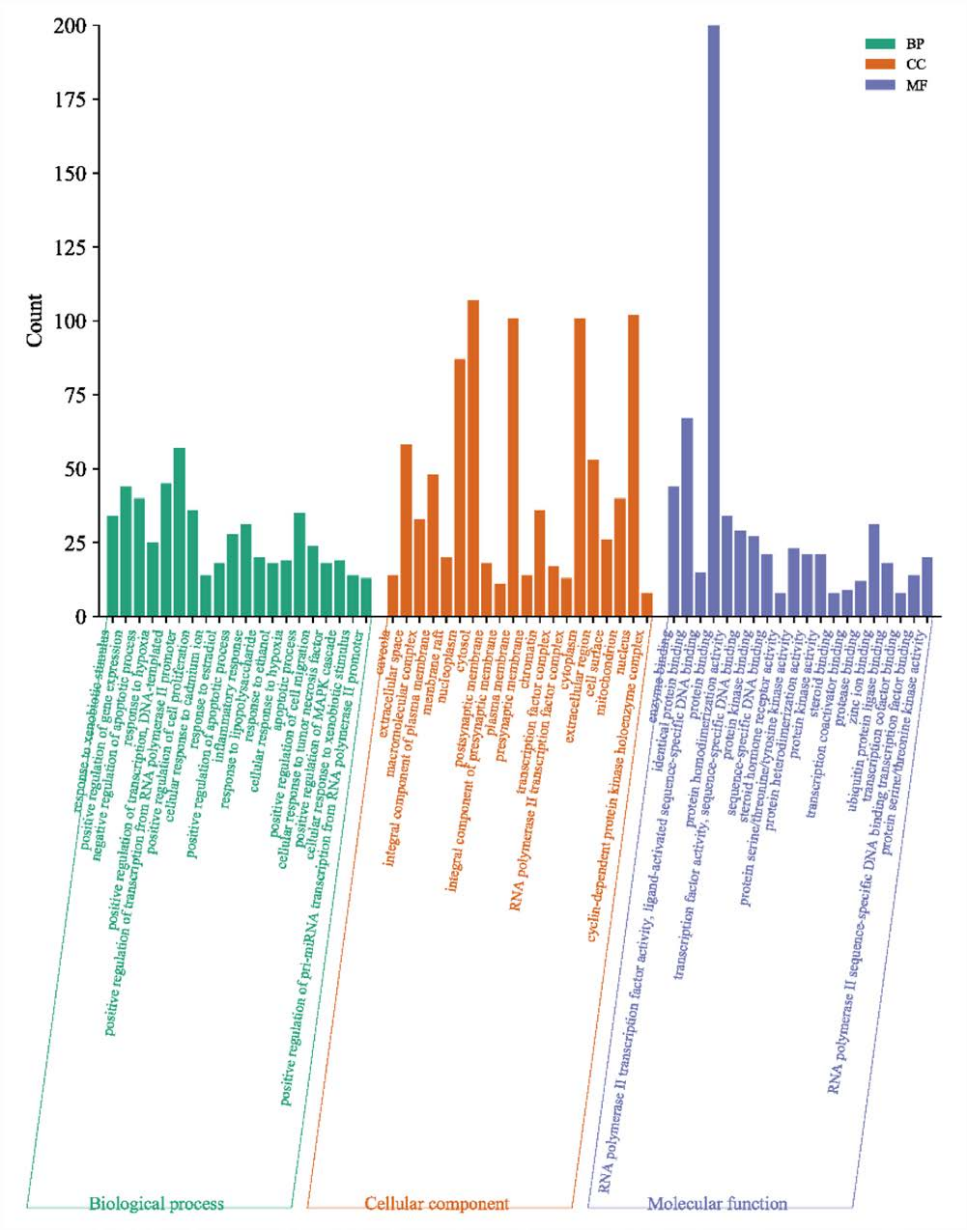
The GO enrichment results. BB = biological process, CC = cellular component, GO = gene ontology, MF = molecular function.

GO enrichment analysis was performed on the acquired 224 significant targets, resulting in a total of 1243 entries for GO enrichment analysis functions. The analysis identified 727 biological processes (GO-BP), including drug response, response to estrogen, response to lipopolysaccharide, inflammatory response, cell response to hypoxia, cell response to tumor necrosis factor-α, cell apoptosis, and others. Additionally, 153 molecular functions (GO-MF) were detected, such as enzyme binding, protein binding, transcription factor, protein heterodimerization activity, steroid hormone receptor activity, steroid binding, protease binding, chromatin binding, and others. The results were complemented by 102 cell components (GO-CC). The top 20 significant entries were selected and are shown in Figure 5.

### 3.7. KEGG enrichment analysis

**Figure 6.**
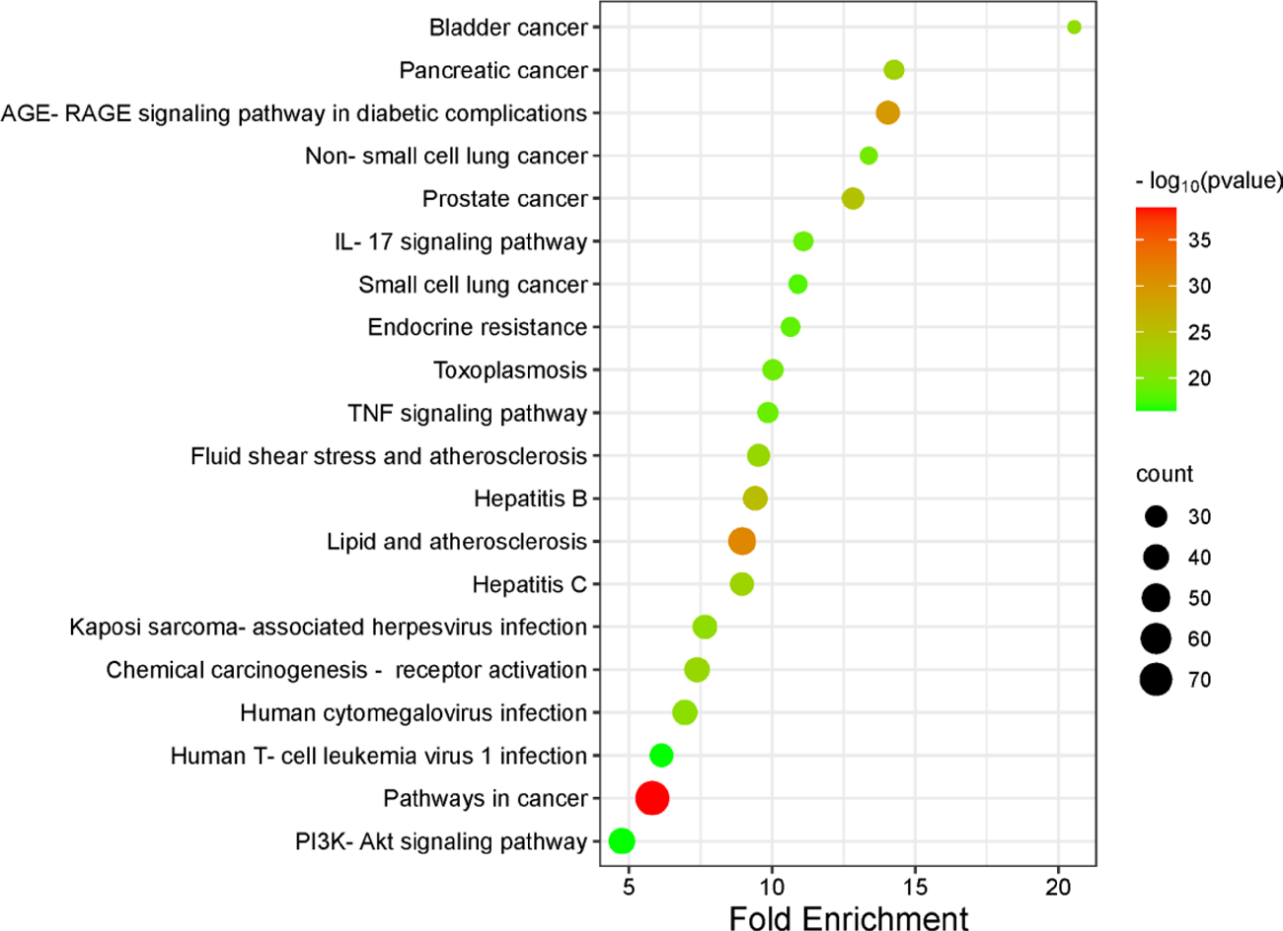
KEGG pathway analysis results bubble chart (top 20 terms). The size of each circle represents the number of enriched genes in the pathway. A larger circle indicates a higher number of enriched genes. Meanwhile, the color depth reflects the *P* value, with a smaller *P* value resulting in a deeper shade of red.

The KEGG enrichment analysis of the 224 key targets yielded 189 enriched pathways. The top 20 pathways were chosen for display based on *P* value ranking. The treatment of DPVD using SMTAD predominantly targets pathways related to cancer, lipid and atherosclerosis, the AGE-RAGE signaling pathway, the interleukin IL-17 signaling pathway, the tumor necrosis factor signaling pathway (TNF), the endocrine resistance pathway, the cellular senescence pathway, and 178 other pathways, as illustrated in Figure 6.

### 3.8. Molecular docking verification

**Figure 7.**
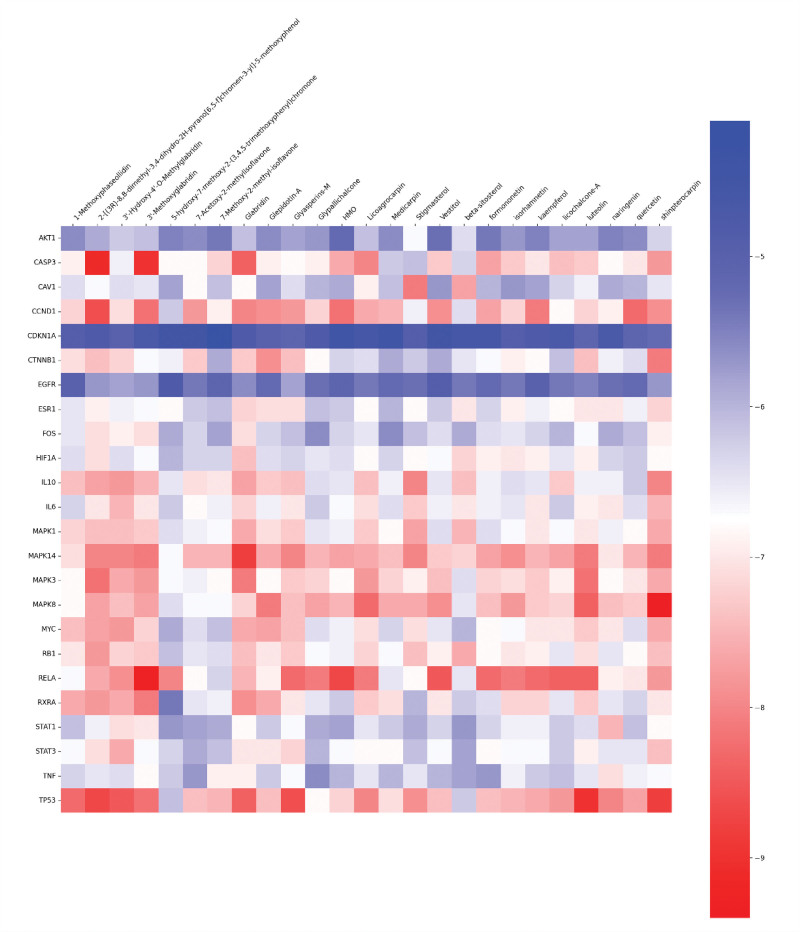
Heat map of molecular docking. Blue indicates that the binding energy is greater than −7 kcal/mol. The darker the shade, the greater the binding energy, and the less binding activity between the active component and the target. Red denotes a binding energy data less than −7 kcal/mol. The darker the shade, the lower the binding energy between the active component and the target, and the higher the binding activity between the active component and the target.

**Figure 8.**
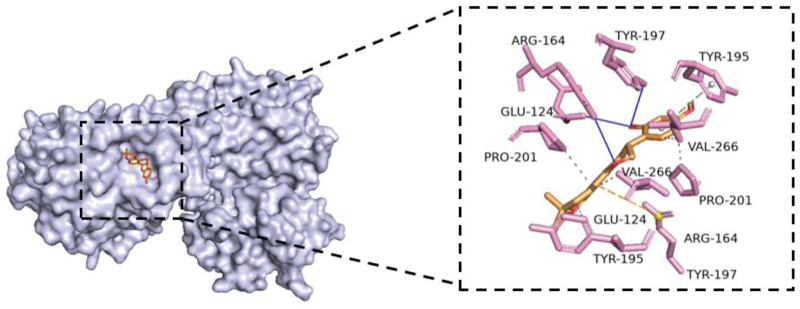
The molecular docking structure of CASP3 and 2-[(3R)-8,8-dimethyl-3,4-dihydro-2H-pyrano[6,5-f]chromen-3-yl]-5-methoxyphenol. The docking energy of CASP3 and 2-[(3R)-8,8-dimethyl-3,4-dihydro-2H-pyrano[6,5-f]chromen-3-yl]-5-methoxyphenol is −9.1 kcal/mol. Being lower than −5 kcal/mol, it indicates that CASP3 and 2-[(3R)-8,8-dimethyl-3,4-dihydro-2H-pyrano[6,5-f]chromen-3-yl]-5-methoxyphenol have a strong affinity. 2-[(3R)-8,8-dimethyl-3,4-dihydro-2H-pyrano[6,5-f]chromen-3-yl]-5-methoxyphenol occupies the active cavity formed by the CASP3 protein residues (TYR, PRO, VAL, GLU, GLY, and ARG), forming hydrophobic interactions with TYR195, PRO-201, and VAL266; forming hydrogen bonds between TYR-197, ARG-164, GLU-124; π-cation interactions with ARG-164; and π-π stacking with TYR-195.

**Figure 9.**
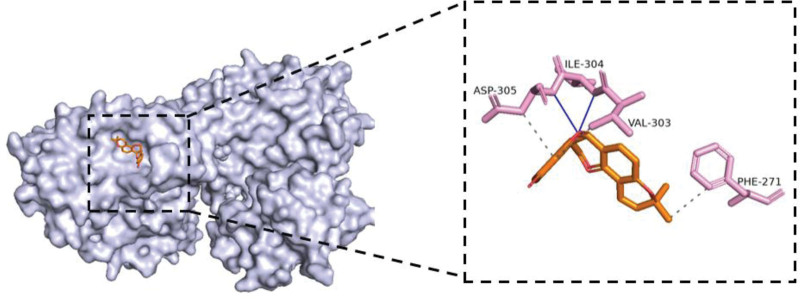
The molecular docking structure of MAPK8 and shinpterocarpin. The docking energy of MAPK8 and shinpterocarpin is −9.4 kcal/mol. Being lower than −5 kcal/mol, it indicates that MAPK8 and shinpterocarpin have a strong affinity. Shinpterocarpin occupies the active cavity formed by the MAPK8 protein residues (ILE, PHE, ASP, and VAL), forming hydrophobic interactions with ASP-305, PHE-271, and VAL-303; forming hydrogen bonds between ILE-304 and ASP-305.

**Figure 10.**
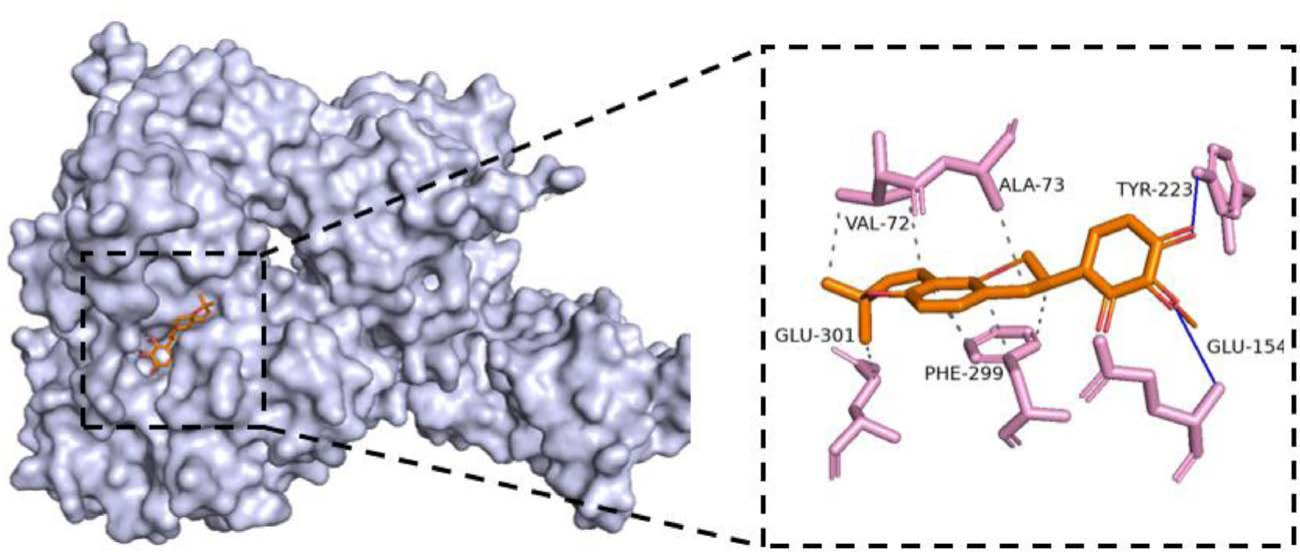
The molecular docking structure of RELA and 3′-Methoxyglabridin. The docking energy of RELA and 3′-Methoxyglabridin is −9.3 kcal/mol. Being lower than −5 kcal/mol, it indicates that RELA and 3′-Methoxyglabridin have a strong affinity. 3′-Methoxyglabridin occupies the active cavity formed by the RELA protein residues (ALA, PHE, GLU, VAL, and TYR), forming hydrophobic interactions with ALA-73, PHE-299, GLU-301, and VAL-72; forming hydrogen bonds between GLU-154 and TYR-223.

The study entailed selecting 25 key effective components and 24 potential core target proteins for molecular docking using AutoDock Vina. This involved calculating the binding energy of each core component with its corresponding target protein. Figure 7 displays a heatmap that was generated using the matplotlib package in Python. If the binding energy between an active compound and a target protein receptor is less than −5 kcal/mol, it is considered to have good binding activity, and if it is less than −7 kcal/mol, it is considered to have strong binding activity.^[18]^ For the molecular docking structure display, the parts with smaller binding energy were selected and are shown in Figures 8–10.

## 4. Discussion

The treatment of Diabetic Peripheral Vascular Disease (DPVD) with traditional Chinese medicine has a long history, with traditional Chinese medicine referring to DPVD as “gangrene” or “pulse paralysis.” The “Collection of Insights on Sores” states: “^[25]^Gangrene is a disease where sores form on the toes, in severe cases they ulcerate and turn purple-black, without pain or itch, and over time the joints fall off, hence the name... Initially it appears like millet, a yellow blister, the skin color is dark purple, like a cooked red date, the black qi spreads, the rot gradually opens, the fingers infect each other, even to the extent of the foot surface hurting like being scalded by boiling water, the stench is unbearable, thus becoming a case of ‘five failures’.” The main types of medical certificates are damp-heat toxin excess type, blood stasis obstruction type, and heat-toxin injuring yin type.^[26]^ The treatment should clear heat and detoxify, promote blood circulation and relieve pain. The “New Compilation of Proven Prescriptions” records^[27]^: “This gangrene occurs on the fingers of the hands and feet, or on the fingertips, or on the finger joints, finger gaps. Initially, it may be white and extremely painful, or a yellow blister like a grain of millet may appear. The skin may be like a cooked red date, the black color does not recede, and after a long time it ulcerates, the joints fall off one by one, extending to the back of the hand and foot rotting and blackening, the pain is unbearable... Then use honeysuckle, Radix Scrophulariae each 3 liang, Angelica 2 liang, licorice one liang, decocted in water for oral administration.” SMYAD is a classic famous prescription for treating gangrene, with only 4 medicinal ingredients, its efficacy is extraordinary, the dosage is large and the power is focused, it is often used for gangrene ulceration, when heat-toxin is flourishing and yin-blood is injured, the therapeutic effect is significant, it has been selected into the “Directory of Ancient Classic Famous Prescriptions (First Batch)” released by the National Administration of Traditional Chinese Medicine on April 16, 2018, becoming one of the 100 classic famous prescriptions.^[28]^ However, with prolonged use and high doses, a small number of patients may experience side effects such as nausea and vomiting. It still needs to be added or subtracted according to the syndrome, such as when damp-heat is heavy, add Phellodendron, Atractylodes, Anemarrhena, Alisma; when blood stasis is obvious, add Peach Kernel, Safflower, Polygonum; when qi and blood are both deficient, add Codonopsis, Astragalus, Rehmannia, Atractylodes, Chicken Blood Vine, etc. Modern pharmacological studies have found that SMYAD can effectively reduce the levels of inflammatory factors such as TNF-α, IL-1β, and IL-6, inhibit the expression of chemokines such as CXCL1, CXCL2, and IL-8,^[6]^ promote the apoptosis of neutrophils, reduce the production of ROS and NETs, and improve the inflammatory response. It can also inhibit thrombosis formation and anti-inflammatory effects by regulating various metabolic pathways such as cysteine and methionine metabolism, phenylalanine metabolism, tyrosine metabolism, and vitamin B6 metabolism.^[29]^

Based on network pharmacology methods, a total of 25 active ingredients, including quercetin, kaempferol, luteolin, naringenin, and β-sitosterol, are widely distributed in various medicinal herbs in SMYAD, and many of these components have been scientifically proven to have biological effects. Quercetin, kaempferol, and luteolin are flavonoid compounds that function by lowering blood sugar, reducing lipids, having anti-inflammatory effects, and protecting vascular endothelial cells. Quercetin has been shown to reduce the expression of pro-inflammatory cytokines IL-1β and TNF-α, as well as PGE 2 and LTB-4 in animal serum. In addition, it can increase the level of anti-inflammatory cytokine IL-10 in serum and non-enzymatic antioxidant glutathione in serum, which exerts anti-inflammatory effects.^[30]^ It can reduce macrophage foam cell formation and improve atherosclerosis by increasing macrophage CYP27A1 expression, inhibiting CD36-mediated cholesterol uptake, and enhancing cholesterol efflux mediated by the LXRα-ABCA1/G1 signaling pathway.^[31]^ Rutoside not only alleviates the inflammatory response of sepsis induced by LPS and stabilizes the vascular endothelial barrier by regulating the SphK1/S1P signaling pathway, but it also promotes cell migration and angiogenesis by regulating the protein expression of VEGFA and MMP2.^[32–34]^ Hesperidin reduces inflammation and apoptosis in mouse cardiomyocytes induced by LPS by downregulating Nlrp3.^[35]^ Naringin has the potential to improve the levels of sugar and lipid metabolism in diabetic mice. It can also activate Nrf2 activity to promote the activity of related phase II detoxifying enzymes and reduce vascular intimal inflammatory infiltration by inhibiting ferroptosis. Consequently, Naringin can prove beneficial in improving diabetic vascular endothelial injury.^[36,37]^ β-Sitosterol is a plant sterol, a secondary metabolite found in organisms. It has been shown to possess various physiological activities, including antibacterial, anti-inflammatory, antioxidant, anti-atherosclerosis, lipid-lowering, anti-aging, and immune regulation functions. Consequently, it finds widespread use in the clinical treatment of conditions such as cardiovascular diseases, diabetes, and immune diseases.^[38,39]^ Studies have confirmed the efficacy of β-sitosterol in reducing mouse foot swelling and ear edema, as well as reducing the neutrophil count in peripheral blood.^[40]^ Beta-sitosterol has been shown to lower plasma total cholesterol, oxidized low-density lipoprotein, and C-reactive protein. Some researchers believe that it may improve the area of atherosclerotic lesions at the base of the aorta by inhibiting the expression of vascular cell adhesion molecule-1 and preventing the adhesion of THP-1 monocytes to vascular smooth muscle cells.^[41]^ However, further research is needed to confirm these findings. Among them, quercetin can reduce the expression of pro-inflammatory cytokines IL-1β and TNF-α, as well as PGE 2 and LTB-4 in animal serum, increase the level of anti-inflammatory cytokine IL-10 in serum, and increase the level of non-enzymatic antioxidant glutathione in serum, thereby achieving an anti-inflammatory effect.

Based on the PPI protein interaction network analysis, SMYAD targets 24 core proteins, such as AKT1, MAPK3, TP53, STAT3, MAPK1, RELA, FOS, CTNNB1, and TNF, that are related to blood vessel reconstruction, cell proliferation and apoptosis, tumors, and lipid metabolism to treat DPVD. In the treatment of peripheral vascular disease, the control of inflammation and the establishment of collateral circulation is crucial in treating peripheral vascular diseases, as it can aid in symptom improvement and disease development.^[42]^ AKT1 is a protein kinase and a major mediator of angiogenic signal transduction. Studies have found that by acting on the Akt and ERK1/2 pathways, it can prevent peripheral vascular inflammation and atherosclerosis in diabetic mice.^[42]^ Research studies have revealed that the skeletal protein ENH accelerates the deactivation of AKT1 in endothelial cells after vascular injury by binding to the phosphatase PHLPP2 and the kinase AKT1, promoting the dephosphorylation of AKT1 in endothelial cells, inhibiting NO production, and achieving the objective of anti-inflammation and fostering vascular reconstruction.^[43]^ Researchers believe that the p38-activated MAPK and ERK1/2 signaling pathways can induce cell inflammation. Inhibiting p38 reduces IL-6 and TNF-α levels, while inhibiting ERK1/2 reduces IL-6 expression.^[44]^ Researchers have validated in mice lacking the MT4-MMP protease that stimulating p38 MAPK activity can enhance arteriogenesis and improve blood supply after peripheral arterial occlusion.^[45]^ FOX protein is a winged helix family of DNA-binding region transcription factors. It plays an important role in carbohydrate and fat metabolism, biological aging and immune regulation, development, and disease in mammals. Among them, TREM1 can promote the recruitment of FOXM1 positive neutrophils, achieving anti-inflammatory and wound healing effects.^[46]^ FOXM1 plays a crucial role in vascular repair mechanisms by restoring vascular integrity, eliminating inflammation, and promoting repair after inflammatory lung injury.^[47]^ Therefore, it is speculated that SMYAD may improve peripheral vascular blood supply and control vascular inflammatory response by regulating the expression of core targets such as AKT1, p38/MAPK, FOXM1, etc.

The KEGG pathway enrichment analysis indicates that SMYAD signaling pathways for treating DPVD primarily involve TNF signaling, AGE-RAGE signaling for diabetes complications, and the IL-17 signaling pathway pathways in cancer, lipid and atherosclerosis, endocrine resistance, cellular senescence, etc. TNF-α is found to influence adhesion factors, leading to vascular inflammation and blood vessel permeability around the lesion.^[48]^ Kaempferol has antihypertensive, cardiovascular protective, antioxidant, and anti-inflammatory effects in NO-dependent hypertensive rats. The potential mechanism of rutin in preventing L-NAME-induced cardiovascular changes is due to inhibition of the TNF-α pathway.^[49]^ AGE, a metabolic byproduct in a high-sugar environment, frequently interacts with the receptor for advanced glycation end products (RAGE). This interaction causes oxidative stress, inflammation, and fibrosis in myocardial and vascular tissue, resulting in a variety of peripheral vascular and cardiovascular issues.^[50]^ IL-17 is a pro-inflammatory factor that is selectively produced by T helper cell 17 (Th17). Increased expression of IL-17 can induce inflammatory factors and chemokines, mediating inflammatory responses that lead to various inflammatory diseases, immune diseases, and tumors. Previous studies have shown that IL-17 and various proteins can cooperate to enhance the expression of chemokines. Activation of the TBK1-HIF-1α-mediated IL-17/IL-10 signal due to high sugar environment may exacerbate vascular atherosclerotic lesions. Therefore, it is hypothesized that SMYAD may prevent and treat DPVD by inhibiting signal molecules in the diabetes complications AGE-RAGE signaling pathway, IL-17 signaling pathway, and TNF pathway.^[51]^ Therefore, it is considered that the mechanism of SMYAD in treating DPVD may be related to the TNF signaling pathway, the AGE-RAGE signaling pathway in diabetes complications, and the IL-17 signaling pathway. The results of molecular docking demonstrate that the 25 active compounds exhibit stable docking structures with the 24 primary target proteins, indicating their functionality. These outcomes support the precision of this research.

## 5. Conclusion

Based on the above research results, it is indicated that quercetin, kaempferol, luteolin, 7-Methoxy-2-methyl isoflavone, naringenin, Formononetin, Isorhamnetin, beta-sitosterol, Licochalcone A, Medicarpin, Stigmasterol, 2-[(3R)-8,8-dimethyl-3,4-dihydro-2H-pyrano[6,5-f]chromen-3-yl]-5-methoxyphenol, shinpterocarpin, Licoagrocarpin, Vestitol, 1-Methoxyphaseollidin, 3′-Methoxyglabridin, 3′-Hydroxy-4′-O-Methylglabridin, HMO, Glabridin, Glypallichalcone, Glyasperins M, 7-Acetoxy-2-methylisoflavone, Glepidotin A, 5-hydroxy-7-methoxy-2-(3,4,5-trimethoxyphenyl) chromone are the main effective components of SMYAD. AKT1, MAPK3, TP53, STAT3, MAPK1, RELA, FOS, CTNNB1, TNF, ESR1, RXRA, MAPK14, IL-6, MYC, EGFR, CCND1, MAPK8, RB1, CAV1, CDKN1A, STAT1, CASP3, HIF1A, IL-10 are the core target proteins for SMYAD treatment of DPVD. The treatment of DPVD by SMYAD mainly involves pathways such as Lipid and atherosclerosis, AGE-RAGE signaling pathway, Interleukin IL-17 signaling pathway, TNF signaling pathway, etc. The docking structure of the active ingredients and the core targets in the study is stable and responds well.

However, there are still some limitations in our current research. The mechanism of SMYAD in treating diabetic peripheral vascular disease in our study is based on the existing updated computer database, without considering the interaction between different components. Secondly, network pharmacology and molecular docking are only preliminary explorations of possible mechanisms, and the predicted results still need to be further verified through pharmacological experiments and clinical trials.

This study initially predicted the active ingredients and potential targets of SMYAD for DPVD through network pharmacology and molecular docking technology, reflecting the multi-component, multi-target, and multi-pathway treatment of DPVD by SMYAD. It lays a foundation for the development of targeted drugs, clinical application, and related basic research in the future.

## Author contributions

**Conceptualization:** Fang Cao, Yongkang Zhang, Yuan Zong, Xia Feng, Junlin Deng, Yemin Cao.

**Data curation:** Fang Cao.

**Formal analysis:** Fang Cao, Yongkang Zhang, Yuan Zong, Yemin Cao.

**Funding acquisition:** Yemin Cao.

**Investigation:** Fang Cao, Yuan Zong, Junlin Deng.

**Methodology:** Yongkang Zhang, Yuzhen Wang, Yemin Cao.

**Resources:** Yuan Zong, Yuzhen Wang, Yemin Cao.

**Supervision:** Yongkang Zhang, Xia Feng, Yemin Cao.

**Validation:** Xia Feng, Junlin Deng, Yemin Cao.

**Visualization:** Fang Cao, Xia Feng.

**Writing – original draft:** Fang Cao.

**Writing – review & editing:** Yemin Cao.
